# A fast wavelet-based functional association analysis replicates several susceptibility loci for birth weight in a Norwegian population

**DOI:** 10.1186/s12864-021-07582-6

**Published:** 2021-05-02

**Authors:** William R. P. Denault, Julia Romanowska, Øyvind Helgeland, Bo Jacobsson, Håkon K. Gjessing, Astanand Jugessur

**Affiliations:** 1 Department of Genetics and Bioinformatics, Norwegian Institute of Public Health, Oslo, Norway; 2 Department of Global Public Health and Primary Care, University of Bergen, Bergen, Norway; 3 Centre for Fertility and Health (CeFH), Norwegian Institute of Public Health, Oslo, Norway; 4 KG Jebsen Center for Diabetes Research, Department of Clinical Science, University of Bergen, Bergen, Norway; 5 Department of Obstetrics and Gynecology, Institute of Clinical Sciences, Sahlgrenska Academy, University of Gothenburg, Gothenburg, Sweden

**Keywords:** Association analysis, GWAS, Wavelet, Birth weight, Polygenic trait

## Abstract

**Background:**

Birth weight (BW) is one of the most widely studied anthropometric traits in humans because of its role in various adult-onset diseases. The number of loci associated with BW has increased dramatically since the advent of whole-genome screening approaches such as genome-wide association studies (GWASes) and meta-analyses of GWASes (GWAMAs). To further contribute to elucidating the genetic architecture of BW, we analyzed a genotyped Norwegian dataset with information on child’s BW (*N*=9,063) using a slightly modified version of a wavelet-based method by Shim and Stephens (2015) called WaveQTL.

**Results:**

WaveQTL uses wavelet regression for regional testing and offers a more flexible functional modeling framework compared to conventional GWAS methods. To further improve WaveQTL, we added a novel feature termed “zooming strategy” to enhance the detection of associations in typically small regions. The modified WaveQTL replicated five out of the 133 loci previously identified by the largest GWAMA of BW to date by Warrington et al. (2019), even though our sample size was 26 times smaller than that study and 18 times smaller than the second largest GWAMA of BW by Horikoshi et al. (2016). In addition, the modified WaveQTL performed better in regions of high LD between SNPs.

**Conclusions:**

This study is the first adaptation of the original WaveQTL method to the analysis of genome-wide genotypic data. Our results highlight the utility of the modified WaveQTL as a complementary tool for identifying loci that might escape detection by conventional genome-wide screening methods due to power issues. An attractive application of the modified WaveQTL would be to select traits from various public GWAS repositories to investigate whether they might benefit from a second analysis.

**Supplementary Information:**

The online version contains supplementary material available at (10.1186/s12864-021-07582-6).

## Introduction

Birth weight (BW) is known to influence a wide variety of adult-onset diseases, particularly cardio-metabolic diseases such as cardiovascular disease and type 2 diabetes. Although the findings from genome-wide association studies (GWASes) have contributed substantially to our understanding of the genetic underpinnings of BW, the genetic variants identified thus far still account for only a small fraction of the total variance attributable to additive genetic effects. For example, the largest genome-wide association meta-analysis (GWAMA) of BW to date [1] showed that only 28.5 percent of the variance in BW could be attributed to genetic variants carried by the fetus. The conundrum of missing heritability is a recurrent theme in genetic studies of complex traits and has spurred widespread interest in investigating more complex disease mechanisms than single-SNP associations, such as parent-of-origin effects, epistasis, and gene-environment interaction effects. It has also inspired the development of alternative methods that are more efficient at capturing more of the variants potentially missed by traditional GWAS methodology.

Based on a recent report [2], a large fraction of the missing heritability could be accounted for by the covariance of genetic effects and by SNPs with very small effects. The current paper focuses on the second part of the problem and investigates the use of regional tests to identify clusters of SNPs with small effects in large genomic regions. Statistical methods that can incorporate multi-marker effects and machine-learning techniques for analyzing genome-wide genotypic data have been available for some time (e.g., see [3] and [4]). These and other multi-marker methods for GWAS, such as the Sequence Kernel Association Test (SKAT) [5] and the Burden test [6], only test for association in small genomic regions ranging from 5 kb to 25 kb [6] and are thus not adequately equipped to exploit the regional effects of larger stretches of the genome.

To address these limitations, Vsevolozhskaya and colleagues [7] explored the use of wavelet-based methods to screen the entire genome for associations and showed that such approaches may improve the power for detecting an association. However, their method was only applicable to dichotomous traits and the analyses were somewhat limited due to the small sample sizes (ranging from 50 to 1000 individuals). Here, we extend the analysis of Vsevolozhskaya and colleagues and perform a GWAS of a continuous trait, BW, on 9,063 individuals using a wavelet-based method. To do so, we take advantage of wavelet-based association methods for quantitative trait locus (QTL) analysis of functional phenotypes [8–10]. In particular, we reverse the standard strategy of methods designed to detect QTL by treating the individual genotypes as functions varying between 0 (homozygous for the major allele), 1 (heterozygous) and 2 (homozygous for the minor allele) across the genome and testing these functions for associations with a univariate phenotype that can be continuous or binary.

For the current analyses, we use the WaveQTL method by Shim and Stephens [9] because it is fast and scales well for genome-wide screening. Although WaveQTL was originally developed to identify SNPs that influence chromatin accessibility, we show that it can easily be adapted to screen for associations between a function and a trait. Specifically, we tailor WaveQTL to enable a genome-wide screening for associations between wavelets and a continuous trait (here, BW). We refer to this extended version of WaveQTL as “modified WaveQTL”, which mainly consists of partitioning the genome into smaller regions of 1 Mb in size and testing each region for association with BW. In addition, we implement a feature termed “zooming strategy” to enhance the detection of associations in typically small regions in order to improve statistical power while controlling for false positives. Using the modified WaveQTL, we perform a GWAS of BW based on genotypes from 9,063 children from the Norwegian Mother, father and Child Cohort Study (MoBa) [11].

## Methods

### Study population and phenotyping

MoBa is an ongoing nationwide pregnancy cohort study [11]. Participants in MoBa were enrolled in the study (1999-2008) from 50 of the 52 hospitals in Norway, and they are predominantly of Caucasian ancestry. Trained nurses at the hospitals measured the children’s birth weight. The genotypes in the MoBa dataset were generated on randomly selected umbilical-cord blood DNA samples (N=11,490) from the MoBa biobank [12]. The exclusion criteria were as follows: stillborn, deceased, twins, and children with missing data in the Medical Birth Registry of Norway (MBRN).

### Materials, genotyping platform, and imputation

11,490 mother-father-newborn trios in the MoBa dataset were genotyped using the Illumina HumanCoreExome BeadChip (San Diego, CA, USA) containing more than 240,000 probes. Principal component (PC) visual checks for ethnicity were performed to remove ethnic outliers. For imputation, we used the Haplotype Reference Consortium (HRC) reference data version HRC.r1.1 (http://www.haplotype-reference-consortium.org/) and the free genotype imputation and phasing service of the Sanger Imputation Server (https://imputation.sanger.ac.uk/). For fast and accurate phasing, the Sanger server uses Positional Burrows-Wheeler Transform (PBWT) for indexing multiple sequence alignments across different genomes [13, 14]. We checked the results of the imputation for consistency by hard-calling markers with an INFO quality score larger than 0.7. Additionally, we checked for Mendelian inconsistencies, excess heterozygosity, deviations from Hardy–Weinberg equilibrium (HWE), and high rates of missingness to ensure that no major technical errors were introduced in the pre-phasing and imputation steps. A total of 7,947,894 SNPs met the following criteria and were included in the current analyses: call rate ≥98*%*, minor allele frequency (MAF) ≥1*%*, and HWE test P ≥10^4^. Samples with a call rate ≤98*%* and with an excess heterozygosity ≥4*SD* were excluded.

### Comparison with other studies

The MoBa dataset used here was included in the largest GWAMA of BW to date by Warrington et al. (2019) [1]. Our results are therefore not independent of the findings reported in that GWAMA. To perform an independent and unbiased comparison, we cross-checked our findings against those of the next largest GWAMA of BW that did not include the MoBa dataset, which is the study by Horikoshi et al. [15]. Horikoshi and colleagues identified 60 genome-wide significant loci in a multi-ancestry sample comprising 153,781 genotyped individuals [15]. In terms of sample size, the MoBa dataset used here is approximately 26 times smaller than the Warrington et al. study [1], 18 times smaller than the Horikoshi et al. study [15], and ten times smaller than another published GWAMA of BW from 2013 [16]. For further validation, we used the MoBa dataset to compare the performance of the modified WaveQTL against the standard methodology used by Horikoshi and colleagues [15].

### Application of modified waveQTL

The original WaveQTL by Shim and Stephens [9] tests for association between an individual function and a covariate of interest using wavelets. Below we provide a brief description of wavelets and the modeling used in WaveQTL. We then provide details of our modified version of WaveQTL.

#### Wavelets and waveQTL modeling

**Fig. 1:**
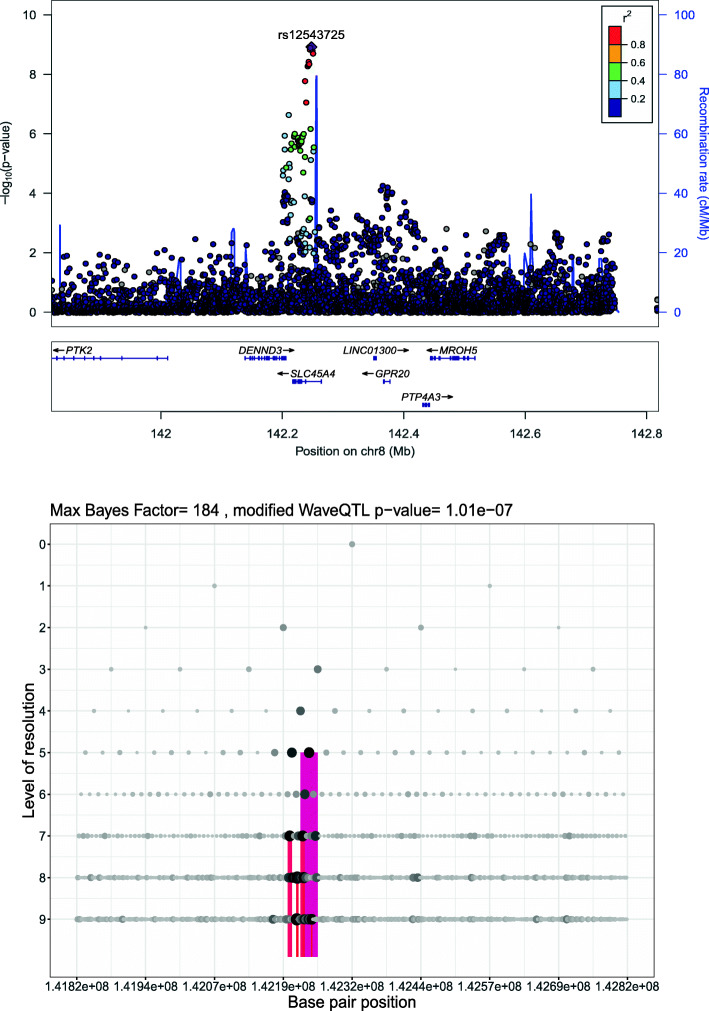
The *SLC45A4* locus detected on chromosome 8. The upper panel is a LocusZoom plot of the locus from the summary data of the Horikoshi et al. study [15]. To ease readability, the maximum number of rows of gene names was truncated to three. LD was computed using the 1000 genomes panel data for a population of European ancestry. The lower panel is the output of the modified WaveQTL for the considered locus, and each dot corresponds to a wavelet coefficient. The size of the dots is proportional to the corresponding Bayes Factor (see Shim and Stephens [9] for details). The regions highlighted in color correspond to the regions contributing to the association

WaveQTL aims at identifying associations between a population of functions and a univariate phenotype *Φ* measured once per function. WaveQTL tests for association between the functions and the trait by testing for association between the wavelet-transformed function and the trait. Wavelets are useful mathematical functions for conducting a Fourier-like transform. There are different types of wavelets [17], and, for the sake of simplicity, we present here only the most straightforward type of wavelet – the Haar wavelet. Like Fourier-transform, wavelet transform allows representing a function as a set of coefficients. The wavelet transform of a function on a given interval is computed via local integrals of the function. The integrals are called wavelet coefficients and are computed for regions of decreasing size, half the size at each step. The wavelet coefficients are indexed using a two-digit code (*s,l*), where the first number, *s*, corresponds to the *scale* or the level of resolution in Fig. 1, while the second number, *l*, corresponds to the location. We refer the reader to a textbook by Nason [18] for a more comprehensive introduction to wavelets and their applications in *R*.

In essence, WaveQTL tests for association between a population of functions and the trait using hierarchical Bayesian modeling that tests (for each wavelet coefficient) whether the trait is associated with the wavelet coefficient, with a prior probability $p(M_{0_{s,l}}) = 1- \pi _{s}$

1 $$\begin{array}{*{20}l} &M_{0}: \tilde{G}_{{sl}} = \beta_{sl,0} +\beta_{sl,C}C+\epsilon  \\ &M_{1}: \tilde{G}_{{sl}} = \beta_{sl,0} + \beta_{sl,1}\Phi +\beta_{sl,C}C+\epsilon \end{array} $$

Where $\tilde {G}_{{sl}}$ is the wavelet coefficient at scale *s* and location *l* based on genotype, *Φ* is the phenotype of interest (here, BW), and C is a confounder. The coefficient *β*_*sl*,1_ can be interpreted as the effect of the phenotype on the wavelet coefficient (sl). Additionally, *π* is a vector of length S, with S being the highest level of resolution. Each component of *π*,*π*_*s*_, represents the proportion of wavelet coefficients at scale *s* associated with *Φ*.

WaveQTL tests for association between the functions and the phenotype by testing the following hypothesis:

2 $$ H_{0}:\pi =(0,...,0) \enspace vs \enspace H_{1}:\exists j \in [0:J], \pi_{j} \ne 0  $$

The significance of *π* is assessed using the following likelihood ratio:

3 $$ \Lambda (\pi, \tilde{X}, \Phi) = \frac{p(\tilde{X} |\pi, \Phi) }{p(\tilde{X} |\pi\equiv 0, \Phi)}  $$

For additional details on how to assess the significance of $\Lambda (\pi, \tilde {X}, \Phi) $, we refer the reader to the original paper by Shim and Stephens [9]. For a fast computation of the *p*-value, we refer the reader to our recent paper [19].

#### Main run of the modified WaveQTL

Here, we treat each individual genotype as a “signal” and BW as the univariate continuous phenotype. For every screened region, we transform the individual genotype into wavelet coefficients and test for association with BW using the WaveQTL framework. An association is identified if the likelihood ratio *p*-value of a region is below the Bonferroni threshold.

In our adaptation of WaveQTL to enable the current GWAS, we used a sliding-window approach to sequentially screen the entire genome for associations. WaveQTL (and, by extension, the modified WaveQTL) is fast and reduces the number of tests to be performed by using overlapping windows of 1 Mb in length. By employing an alternative modeling to the single-SNP linear regression, WaveQTL enhances the detection of associations that are potentially missed by conventional GWAS methodology. The original software implementation of WaveQTL is available at https://github.com/heejungshim/WaveQTL. The modified WaveQTL is distributed as an *R* package on GitHub under the name *mWaveQTL*. The *R* package of the modified WaveQTL includes the zooming strategy (https://github.com/william-denault/mWaveQTL) and a comprehensive example of a typical run.

The user has to specify four parameters to run a GWAS using the modified WaveQTL: i) the region size, ii) the maximum distance between two consecutive SNPs, iii) the level of resolution, and iv) the prior standard deviation for the wavelet effect size. We recommend using half-overlapping regions of 1 Mb as the “region size” parameter and a maximum distance between two consecutive SNPs of 10 kb as the “maximum distance” parameter. Following the recommendations of Zhou and Guan [20], we set the prior standard deviation to $\frac {0.2}{\sqrt (n)}$, where *n* is the number of samples. Based on these criteria, we defined 5,170 regions spanning the entire genome. In addition, the user needs to choose the depth of analysis. In Fig. 1, the y-axis shows how the results of the modified WaveQTL differ by the depth of analysis. As a rule of thumb, we choose ten SNPs per wavelet coefficient for the “level of resolution” parameter. To assign the level of resolution, we set the depth of our analysis to nine. It is important to select an appropriate depth of analysis, because an analysis with insufficient depth might overlook some loci. For example, we observed that most associated regions in the current analysis corresponded to a level of resolution of five or above. These results suggest that using a depth of analysis of four or less would have resulted in not detecting most of the loci.

#### Zooming strategy

One of the main drawbacks of the original WaveQTL is that the sliding-window size is not easily adjustable. If the window is too wide, the signal may be lost in the optimization step due to the large background noise. To overcome this, we developed a “zooming strategy”. As the wavelet coefficients generally remain the same except at the lowest levels, a sub-region can be analyzed using the Bayes factors computed using a larger window size. We implemented the following procedure:

- Detect all the regions that have a Bayes factor above a given threshold (here set to 1).
- For each selected region, extract the sub-region that contains all the Bayes factors above the set threshold.
- Refit the optimization process in Shim and Stephens (2015) [9] on this sub-region to estimate the *p*-value.

A sub-region was considered statistically significant if the associated *p*-value was smaller than $\frac {0.05}{n_{{region}}}\times \frac {size_{sub-region}}{size_{{region}}}$, where *n*_*region*_ is the number of regions initially analyzed (here 5,170). This significance criterion corresponds to the multiple-testing correction for a genome-wide screening based on using regions of the size of the considered sub-region.

## Results

### Application of the modified WaveQTL to BW data

We used the modified WaveQTL to perform a GWAS of approximately eight million SNPs in the imputed MoBa dataset. We assumed an additive genetic model and included sex as a covariate in the analysis. The first ten principal components were also included as covariates to correct for potential population substructure. Overlapping sliding windows of 1 Mb in size were used to analyze a total of 5,170 regions spanning the entire genome. Based on this sliding-window approach, applying a Bonferroni correction for multiple testing led to a significance criterion of *p*≈1×10^−5^. For comparison, we performed a separate GWAS using the traditional additive linear modeling routinely applied to most GWASes, which we simply refer to as “single-SNP modeling”. As with the modified WaveQTL analysis, we assumed an additive genetic model and adjusted for the same set of variables (sex and the first 10 principal components) in the single-SNP modeling. The qq-plot of the *p*-value is displayed in Supplementary Figure 1. Finally, to demonstrate the good calibration of the *p*-values under the null, we performed a GWAS using the modified WaveQTL after permuting the phenotype. The qq-plot of the *p*-values based on this analysis is displayed in Supplementary Figure 2.

**Fig. 2:**
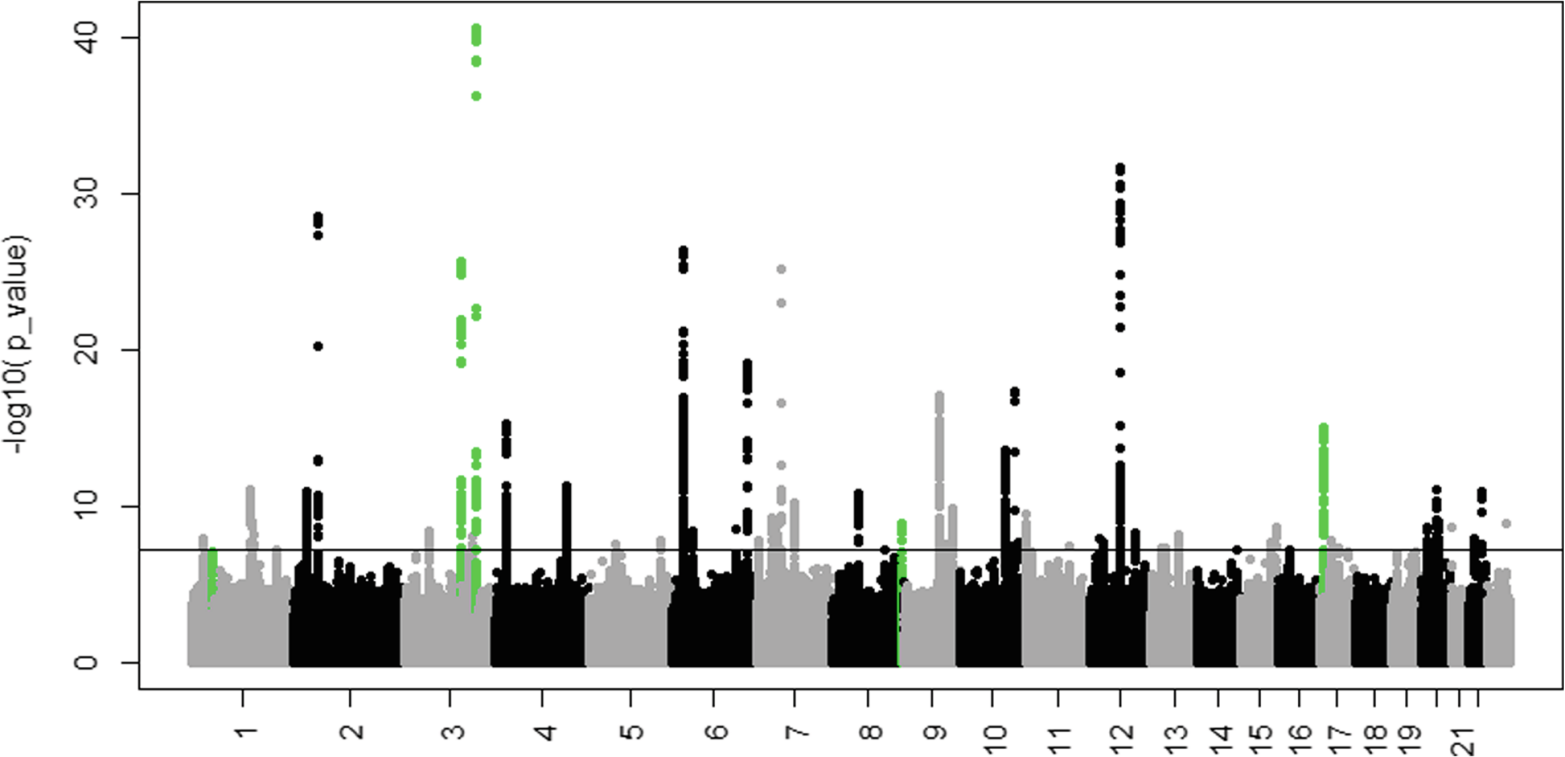
Associations detected by the modified WaveQTL are highlighted in green and are overlaid on the Manhattan plot in the Horikoshi et al. (2016) study (“Extended Data Figure 2” in that paper). The horizontal line indicates the genome-wide significance threshold of 5×10^−8^ for the single-SNP modeling. The corresponding threshold for the main run of the modified WaveQTL is 9.67×10^−6^, as highlighted in Table 1

Table 1 summarizes all the regions in which an association was detected by the modified WaveQTL detected. Figure 2, on the other hand, provides an overview of the results of the modified-WaveQTL in a genome-wide context. The modified WaveQTL initially detected three significant loci for BW, on chromosome 1, 3 and 17 (Supplementary Figure 3-6). We then applied the zooming strategy to offset the possibility that the modified WaveQTL might have missed an association in the optimization step, which may happen if the background noise becomes too large (see Methods for more detail). After applying the new multiple-testing correction for sub-region size in the zoomed analysis, the modified WaveQTL detected two additional loci for BW, on chromosome 3 and 8 (Supplementary Figure 4 and Fig. 1). The single-SNP linear regression did not identify any statistically significant loci in the MoBa dataset (Supplementary Figure 15).

**Table 1:** Summary of the regions detected by the modified WaveQTL

Chr	Start (bp)	End (bp)	Main	*P*-value	Corresponding	Gene name	GWAMA	Sample
			run		correction			size
1	43340639	43403139	Yes	5.06×10^−7^	9.67×10^−6^	*SLC2A1*	[1]	230,069
3	123051305	123133336	Yes	9.81×10^−8^	9.67×10^−6^	*ADCY5*	[21]	27,591
3	156785678	156816928	No	7.00×10^−8^	3.04×10^−7^	*LOC339894/CCNL1*	[16]	61,142
8	142201004	142255692	No	1.01×10^−7^	6.08×10^−7^	*SLC45A4*	[16]	61,142
17	6965237	7215238	Yes	2.82×10^−8^	9.67×10^−6^	*CLDN7/SLC2A4*	[15]	153,781

In the column “Main run”, “Yes” corresponds to a region detected using the modified WaveQTL, and “No” corresponds to a region subsequently detected only after applying the zooming strategy. The column “Corresponding correction” displays the nominal significance level for declaring a region as statistically significant (see Methods for details). The column “GWAMA” corresponds to the GWAMA in which the locus was first detected. The column “Sample Size” corresponds to the sample size of the GWAMA in which the locus was first detected. The genomic coordinates are based on the GRCh37 hg19 genome assembly

All the significantly associated loci detected by the modified WaveQTL have previously been reported in other genetic studies of BW [1, 15, 16, 21]. The gene *ADCY5* on chromosome 3 was identified by the first GWAMA of BW from 2010 [21] in which *n*=27,591 individuals were analyzed; *LOC339894/CCNL1* on chromosome 3 and *SLC45A4* on chromosome 8 were identified by a GWAMA from 2013 (*n*=61,142) [16]; *CLDN7/SCL2A4* on chromosome 17 was identified by a GWAMA from 2016 (*n*=153,781) [15]; and the fifth locus was identified by the currently largest GWAMA of BW by Warrington and co-workers (*n*=230,069) [1]. However, as the Warrington et al. (2019) study included the MoBa dataset used here, the associations detected by the modified WaveQTL are not independent of that study. Table 2 summarizes the overlap between our findings and previous GWAMAs of BW.

**Table 2:** Number of loci detected in previously reported GWAS or GWAMA of birth weight and the overlap with results generated from applying the modified WaveQTL to the MoBa dataset

Sample size	Number of	Overlap	Study name	Year	Reference
	reported loci				
9,063	5	NA	This study	2021	NA
27,591	2	1	Freathy et al.	2010	[21]
61,142	7	3	Horikoshi et al.	2013	[16]
153,781	60	4	Horikoshi et al.	2016	[16]
230,069	190	5	Warrington et al.	2019	[1]

The column “Sample Size” corresponds to the sample size of the GWAS or GWAMA. The column “Number of reported loci” corresponds to the number of loci replicated in each GWAMA. The column “Overlap” corresponds to the number of loci in the GWAMA that overlaps with the five loci reported in our current analyses. The column “Study name” displays the name of the first author for each GWAS or GWAMA and “Year” corresponds to the publication year of the GWAS or GWAMA. All the reported loci in previous GWAMAs have been reported in the largest GWAMA of BW to date by Warrington et al. [1]

### Non-replicated loci

Despite the enhanced statistical power, the modified WaveQTL only detected two of the ten most significant loci in the next largest GWAMA of BW to date, by Horikoshi et al. [15], that did not include our MoBa dataset. Upon closer scrutiny of the 10 most significant loci in the discovery panel in Horikoshi et al. [15], three plausible scenarios emerge as to why eight of the loci might have escaped detection by our approach. The first describes the situation where there is no association signal in the MoBa dataset. This, for example, appears to be the case with the loci neighbouring the genes *AC016696.1* (rs17034876), *LCORL* (rs4144829), *PTCH1*, and *HMGA2* (rs1351394) located on chromosome 2, 4, 9 and 12, respectively (see Supplementary Figure 7, 8, 12 and 14). We found no clear evidence of an association with these loci in the modified WaveQTL analysis.

The second scenario pertains to loci in which the signals are too weak to overcome the multiple-testing burden in the current dataset but that might attain significance in a larger dataset. This might be the case with the loci neighbouring the genes *ESR1* (rs10872678) and *ADBR1* (rs740746) on chromosome 6 and 10, respectively (Supplementary Figure 9 and 13). It is also important to note that the MoBa dataset is roughly 18 times smaller than the one in Horikoshi et al. [15]. Moreover, the *p*-values used for comparison were not corrected for the winner’s curse, where initial studies tend to overestimate the true genetic effect size. The relatively small sample size of the MoBa dataset makes it difficult to distinguish whether the lack of detection in the first scenario was purely due to a cohort-specific effect or due to the winner’s curse.

The third scenario corresponds to the case where the association is only with a single SNP. This is exemplified by the sparse signal observed at *YTK6* (rs138715366) on chromosome 7 (Supplementary Figure 11). Thus, a regional test to detect an association signal from a single SNP that does not show any linkage disequilibrium (LD) with neighboring SNPs might not have sufficient power for detection.

## Discussion

This study is the first adaptation of the method originally described by Shim and Stephens [9] to the analysis of genome-wide genotypic data. The replication of several established loci for BW, even in a sample size 18 and 26 times smaller than those of the two largest GWAMAs of BW to date [1, 15], suggests that the modified WaveQTL may be able to detect genetic associations that are not detectable by a conventional GWAS of the same sample size. As a case in point, the locus Solute carrier family 2 member 1 (*SLC2A1*) was only identified by the modified WaveQTL (*p*=7.3×10^−6^) and the largest GWAMA of BW to date comprising 230,069 individuals [1]. It should be noted, however, that the GWAMA by Warrington and colleagues also included the MoBa dataset; thus, the results of these two studies are not independent of each other. The standard single-SNP linear regression based on an additive model did not detect any genome-wide significant loci for BW in our dataset. This was not unexpected, considering the modest sample size of the MoBa dataset and the known small effect sizes of the SNPs on BW (Supplementary Figure 15).

The ability of the modified WaveQTL to detect associations even with a relatively modest sample size suggests that it may be particularly useful for screening rare diseases where it is inherently difficult to generate a sufficiently large sample size suitable for genome-wide screening. Moreover, the gain in power may be particularly advantageous when examining different subgroups of a disease that are likely to have distinct etiologies (e.g., type 1 and type 2 diabetes [22, 23]). As subgroup analyses further reduce the sample size, the modified WaveQTL may help to offset this limitation by offering a higher statistical power than the regular single-SNP modeling. The modified WaveQTL may thus serve as an initial screening tool for detecting regions harboring significant hits. After a general screening, standard approaches based on a more intuitive and interpretable output, such as polygenic risk scores, can then be applied for downstream fine-mapping efforts.

The modified WaveQTL performed better in regions of high LD between SNPs, as opposed to regions in which only a few SNPs are in strong LD with one another. This is illustrated by the loci *CHR7* and *YKT6-GCK* (Supplementary Figure 11). As most of the loci detected by GWAS or GWAMA exhibit the classical peaks of *p*-values that are characteristic of local LD, our modified WaveQTL is expected to perform better for these types of regions. An obvious application of the modified WaveQTL is to reappraise previously published GWASes to verify whether some of the loci that might have escaped detection by conventional GWAS methodology attain statistical significance with the modified WaveQTL. The publicly accessible GWAS catalog maintained by the European Bioinformatics Institute (EBI; https://www.ebi.ac.uk/gwas/; [24]) and the database of Genotypes and Phenotypes (dbGaP) maintained by the National Center for Biotechnology Information (NCBI; https://www.ncbi.nlm.nih.gov/gap/;[25]) are excellent resources for selecting traits that might benefit from a second analysis.

## Supplementary Information

**Additional file 1** Supplementary figures.

## Data Availability

The full ***R*** package for the modified WaveQTL is freely available on GitHub (https://github.com/william-denault/ffw), and a comprehensive example run of the package is provided in the help function *ffw*. The data can be accessed by sending an application to the Norwegian Regional Committees for Medical and Health Research Ethics (REK, https://helseforskning.etikkom.no/forside?_ikbLanguageCode=us), and upon approval, data administrators at MoBa (https://www.fhi.no/studier/moba/) need to be contacted before secured access to the data can be granted to successful applicants. Declarations

## Abbreviations

BW: Birth weight
GWAS: Genome-wide association study
GWAMA: Genome-wide association meta-analysis
SNP: Single-nucleotide polymorphism
SKAT: Sequence Kernel Association Test
MoBa: Norwegian Mother, Father and Child Cohort Study
MBRN: Medical Birth Registry of Norway
PC: Principal component
HRC: Haplotype reference consortium
PBWT: Positional Burrows-Wheeler Transform
LD: Linkage disequilibrium
MAF: Minor allele frequency
HWE: Hardy-Weinberg equilibrium
