# Supplementary material for: A fast wavelet-based functional association analysis replicates several susceptibility loci for birth weight in a Norwegian population
Source: BMC Genomics. 2021 May 2;22:321. doi: 10.1186/s12864-021-07582-6 (PMC8088671; doi:10.1186/s12864-021-07582-6)
Supplement: Supplementary file 1 — Additional file 1 Supplementary figures. [file 12864_2021_7582_MOESM1_ESM.pdf]

# A fast wavelet-based functional association analysis replicates several susceptibility loci for birth weight in a Norwegian population

William R.P. Denault, Julia Romanowska, Øyvind Helgeland,
Bo Jacobsson, Håkon K. Gjessing, and Astanand Jugessur

## References

[HBD+16] Momoko Horikoshi, Robin N. Beaumont, Felix R. Day, et al. Genome-wide associations for birth weight and correlations with adult disease. *Nature*, 538(7624):248–252, October 2016.

[SS15] Heejung Shim and Matthew Stephens. Wavelet-based genetic association analysis of functional phenotypes arising from high-throughput sequencing assays. *The Annals of Applied Statistics*, 9(2):665–686, June 2015.

## Supplementary figures

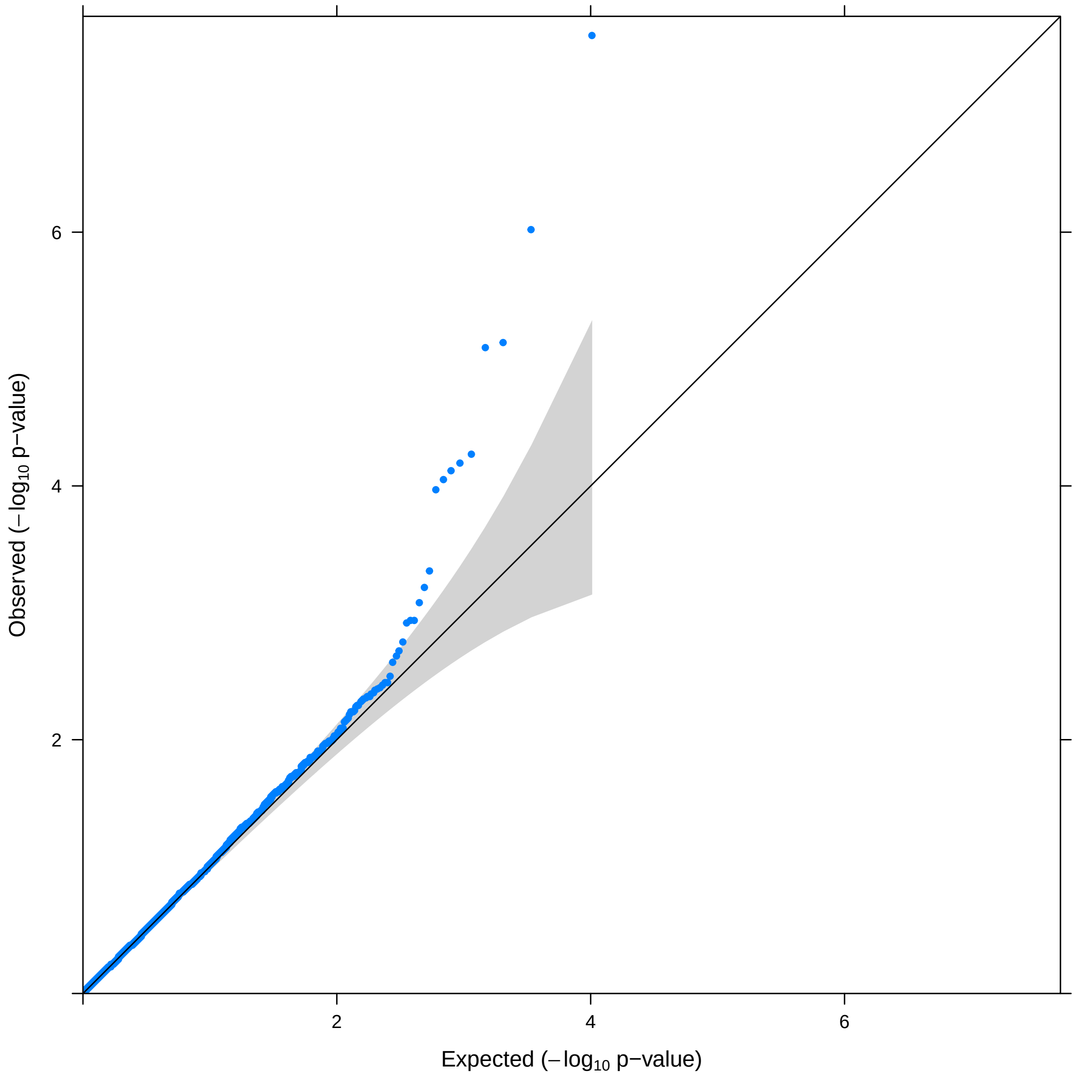


Figure 1: QQplot of the p-values from the GWAS of birth weight based on the modified WaveQTL

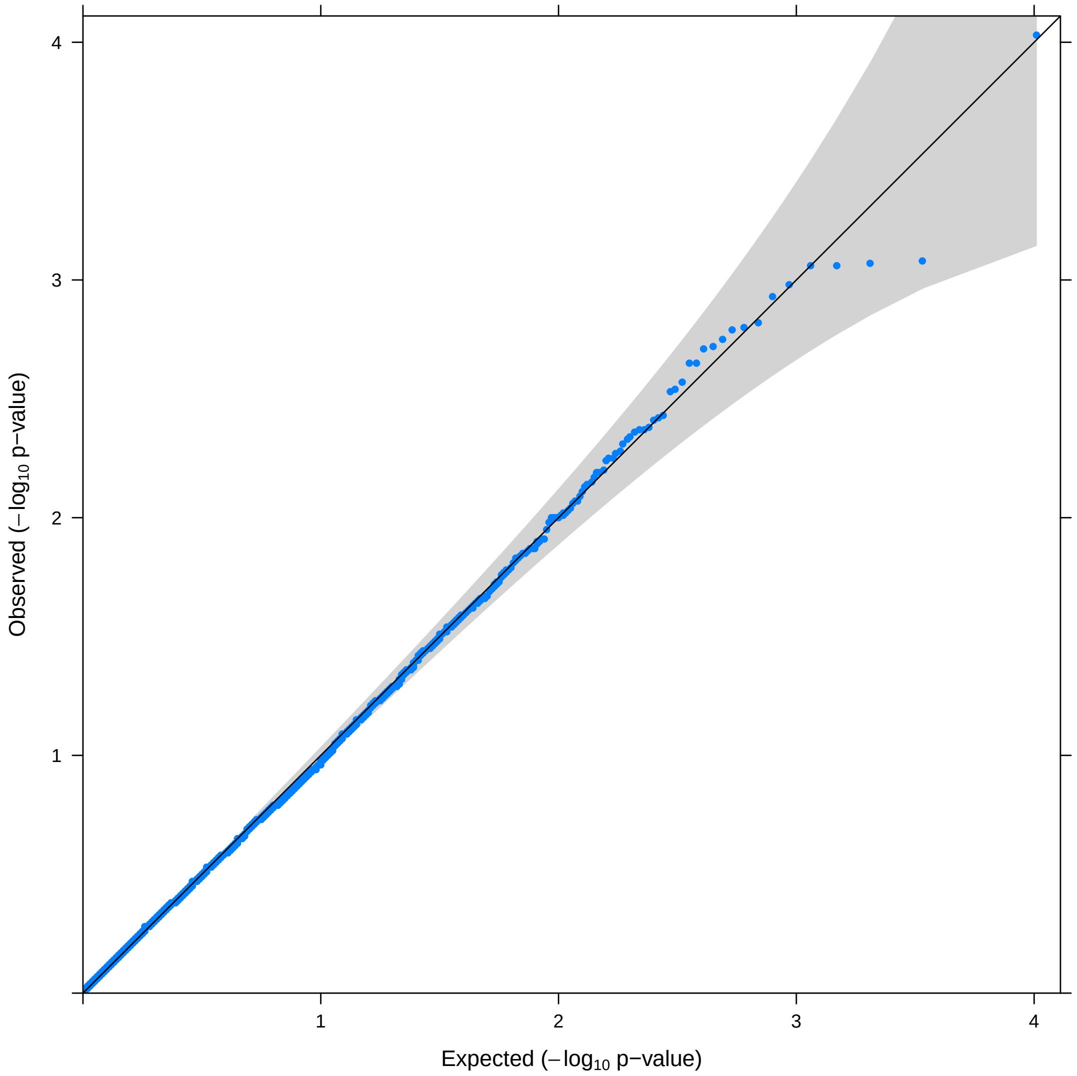


Figure 2: QQplot of the p-values from the GWAS based on the modified WaveQTL using a permutation of the birth weight.

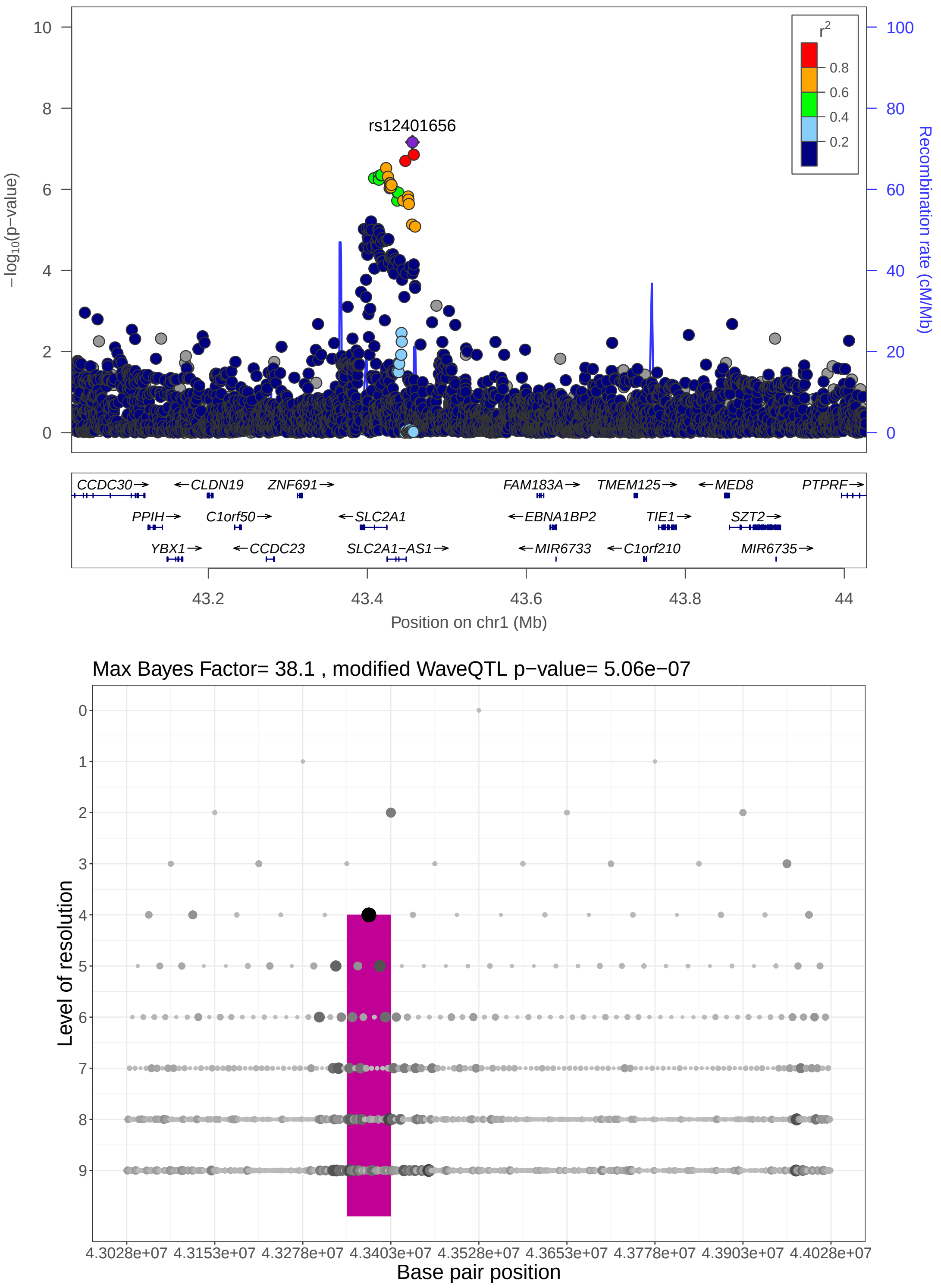


Figure 3: The *SLC2A1* locus detected on chromosome 1. The upper panel is a LocusZoom plot of the locus from the summary data from the Horikoshi *et al.* paper [HBD+16]. To ease readability, the maximum number of rows for gene names was limited to 3. LD was computed using the 1000 genomes panel data for a population of European ancestry. The lower panel is the output of the modified WaveQTL for the considered locus, and each dot represents a wavelet coefficient. The size of the dots is proportional to the corresponding Bayes Factor (see Shim and Stephens [SS15] for further details). The regions highlighted in color correspond to the regions contributing to the association.

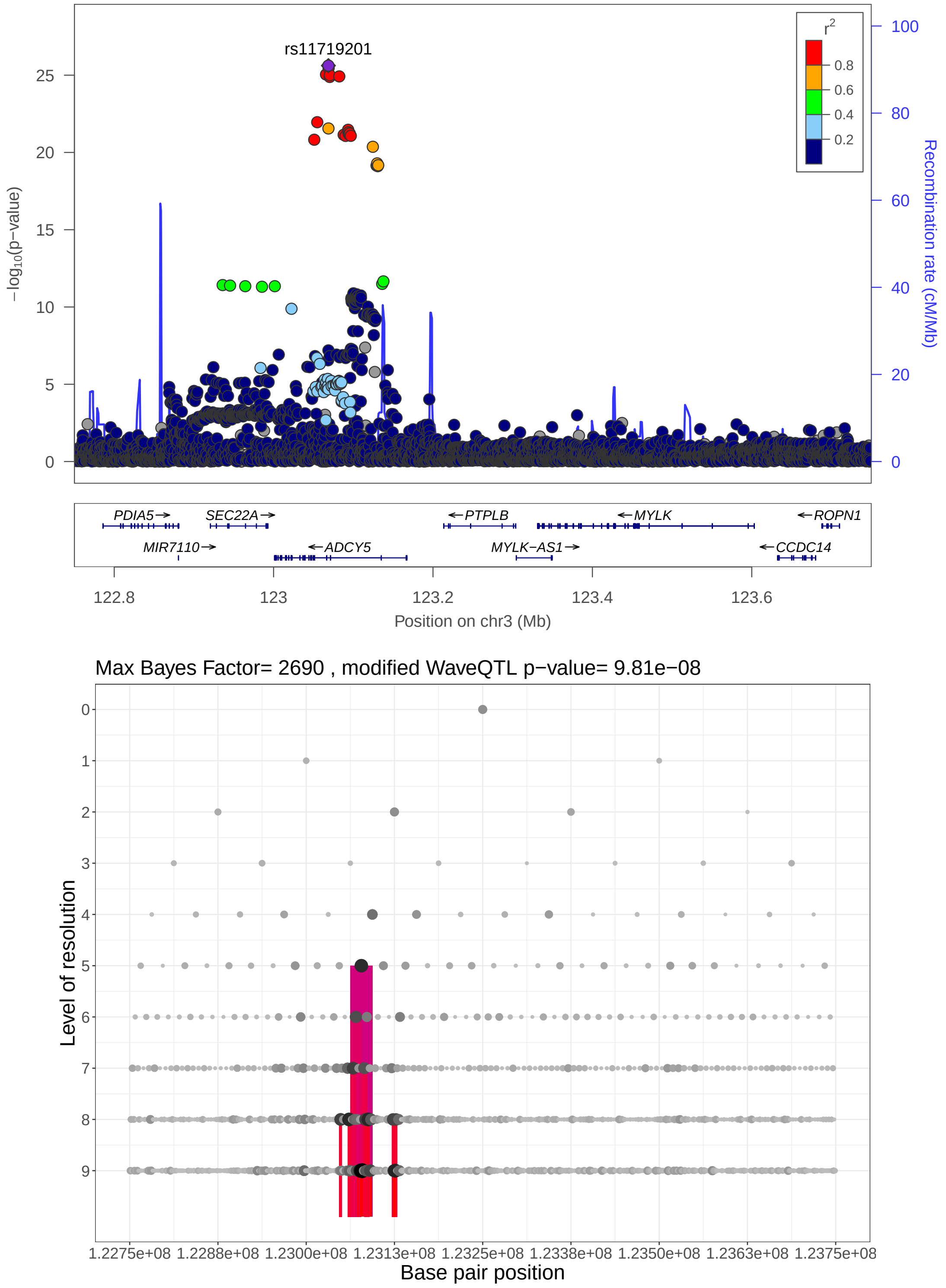


Figure 4: The *ADCY5* locus detected on chromosome 3. The upper panel is a LocusZoom plot of the locus from the summary data from the Horikoshi *et al.* paper [HBD+16].

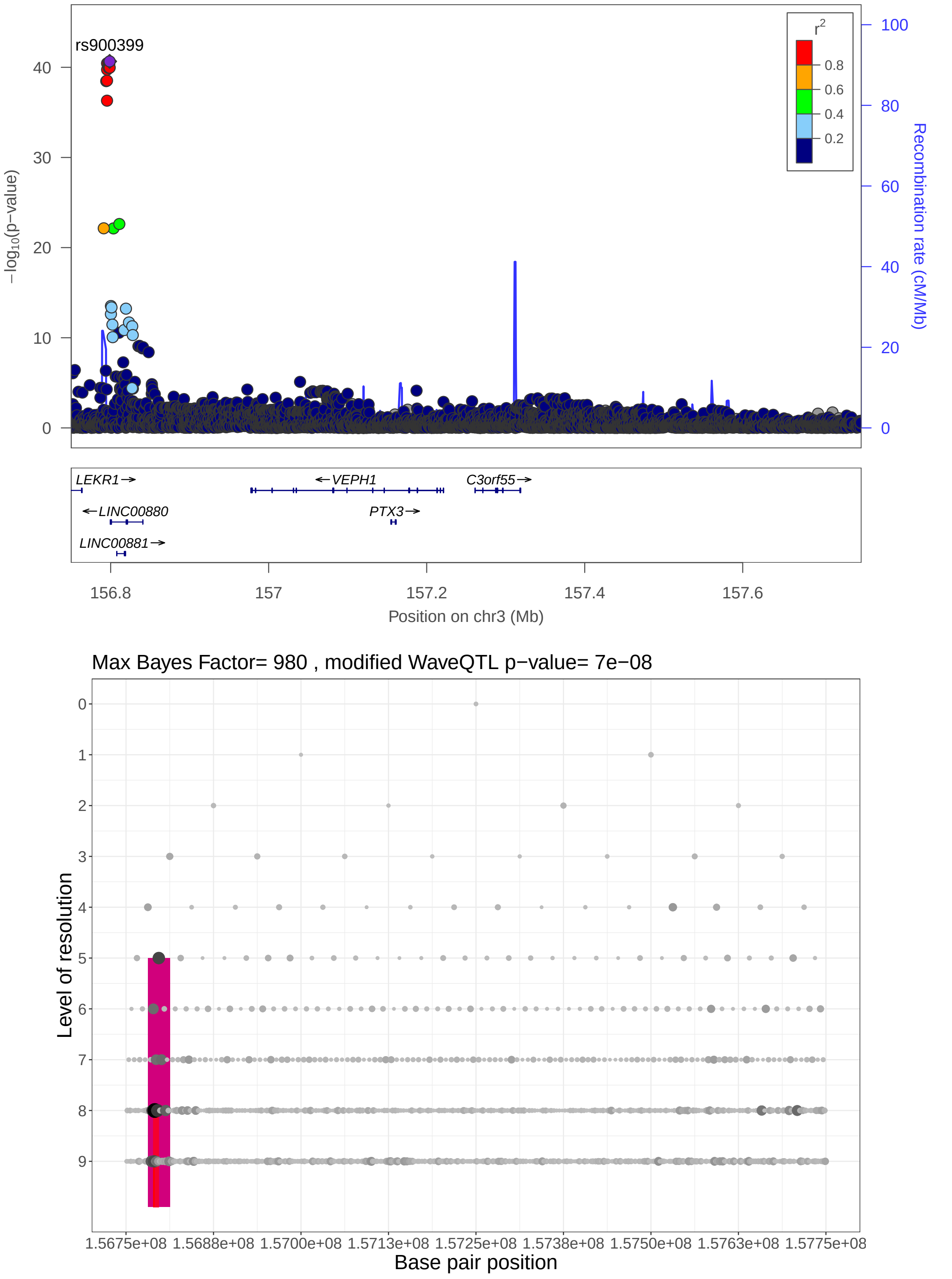


Figure 5: The *LOC339894/CCNL1* locus detected on chromosome 3. The upper panel is a LocusZoom plot of the locus from the summary data from the Horikoshi *et al.* paper [HBD+16].

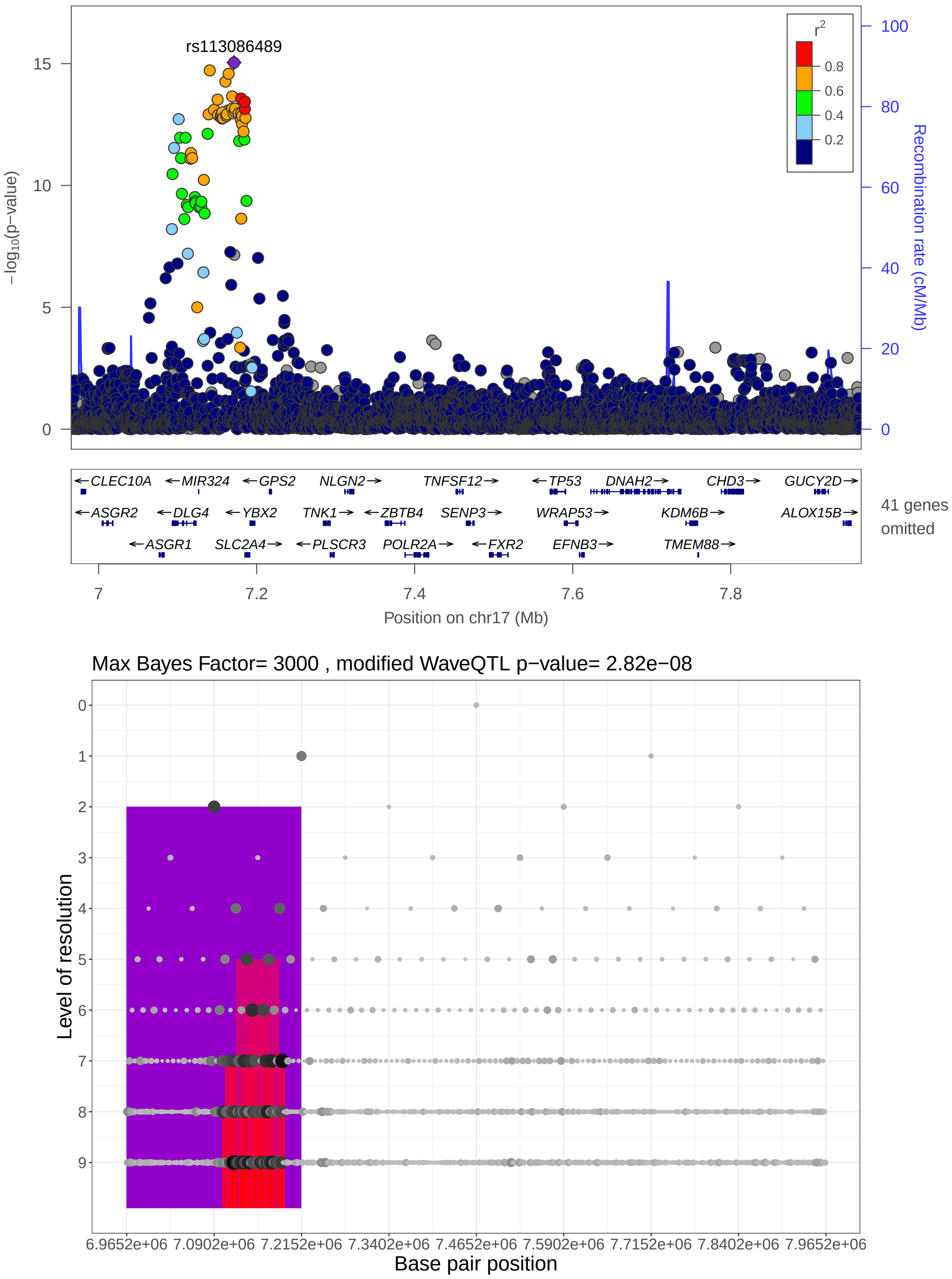


Figure 6: The *CLDN7/SLC2A4* locus detected on chromosome 17. The upper panel is a LocusZoom plot of the locus from the summary data from the Horikoshi *et al.* paper [HBD+16].

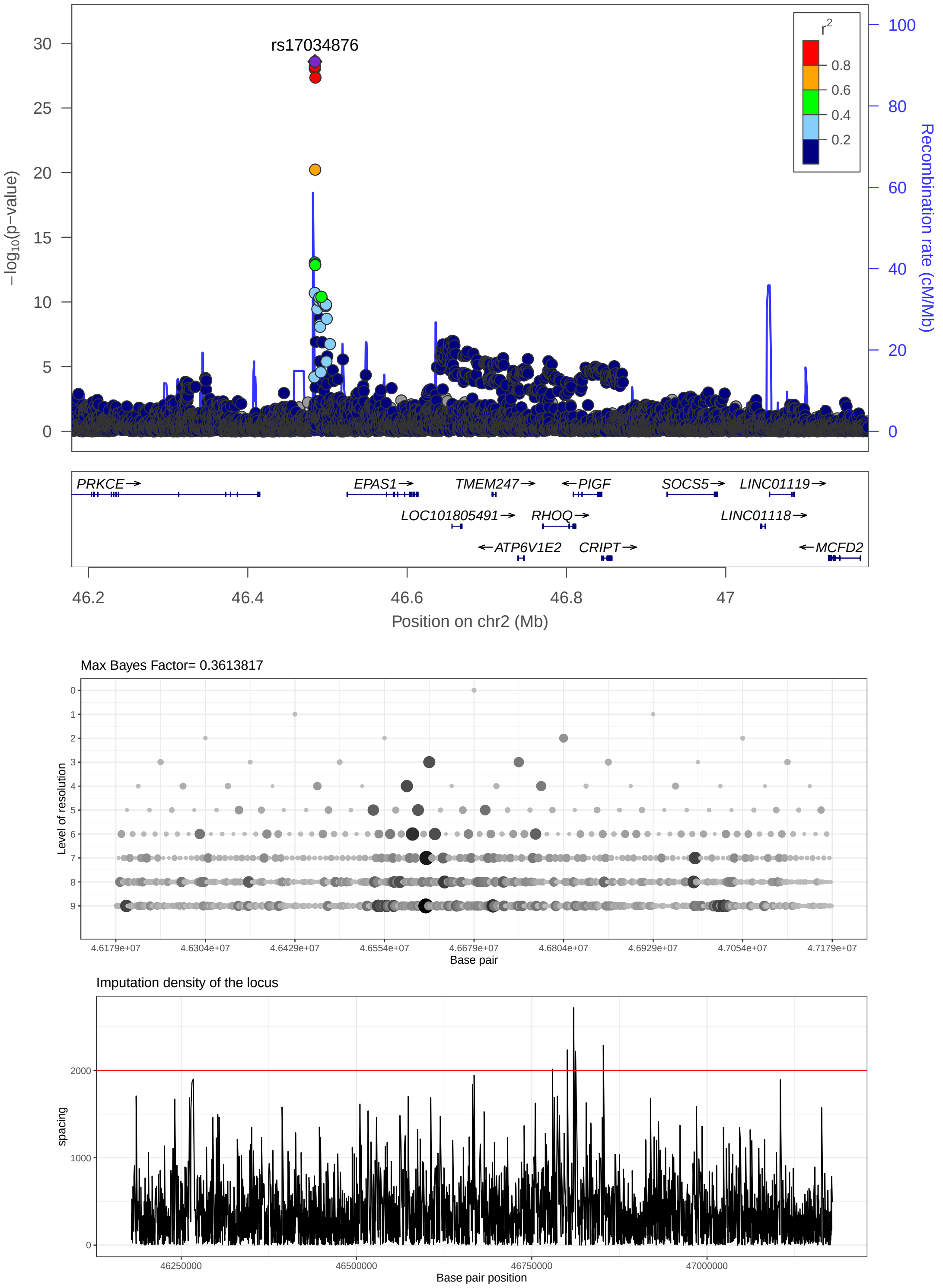


Figure 7: The *AC016696* locus not detected on chromosome 2. The upper panel is a LocusZoom plot of the locus from the summary data from the Horikoshi *et al.* paper [HBD+16].

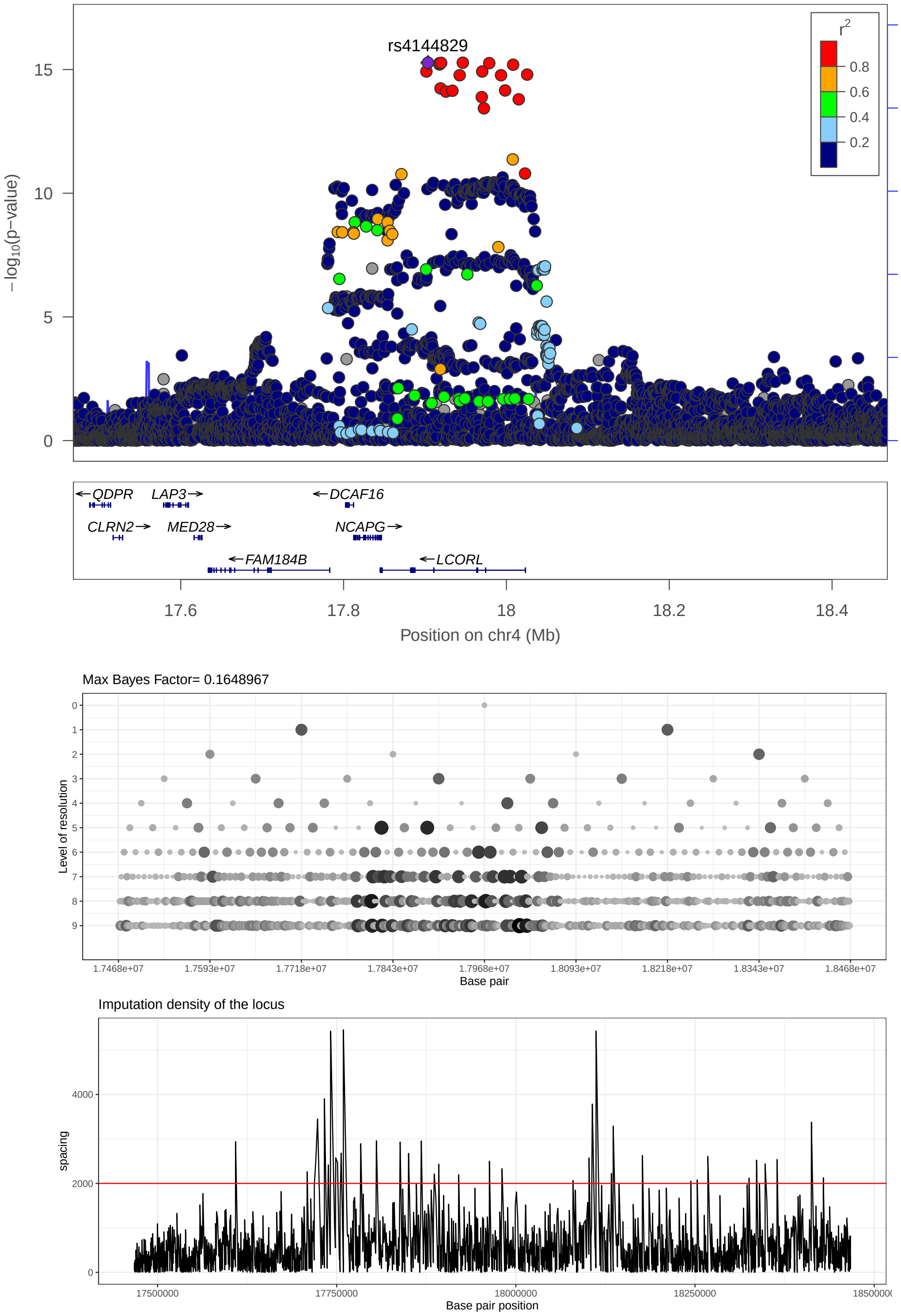


Figure 8: The *LCORL* locus not detected on chromosome 4. The upper panel is a LocusZoom plot of the locus from the summary data from the Horikoshi *et al.* paper [HBD+16].

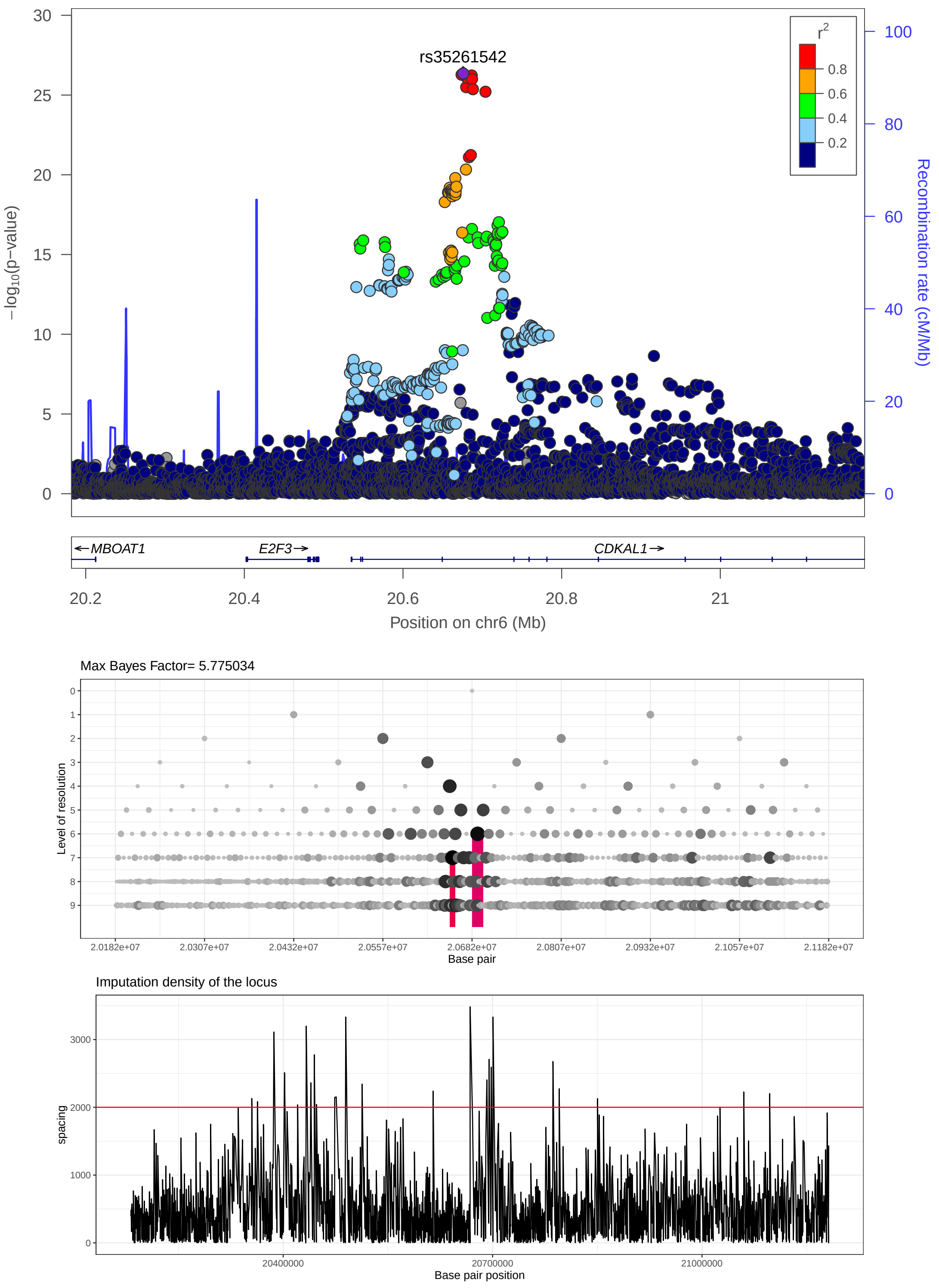


Figure 9: The *CDKAL1* locus not detected on chromosome 6. The upper panel is a LocusZoom plot of the locus from the summary data from the Horikoshi *et al.* paper [HBD+16].

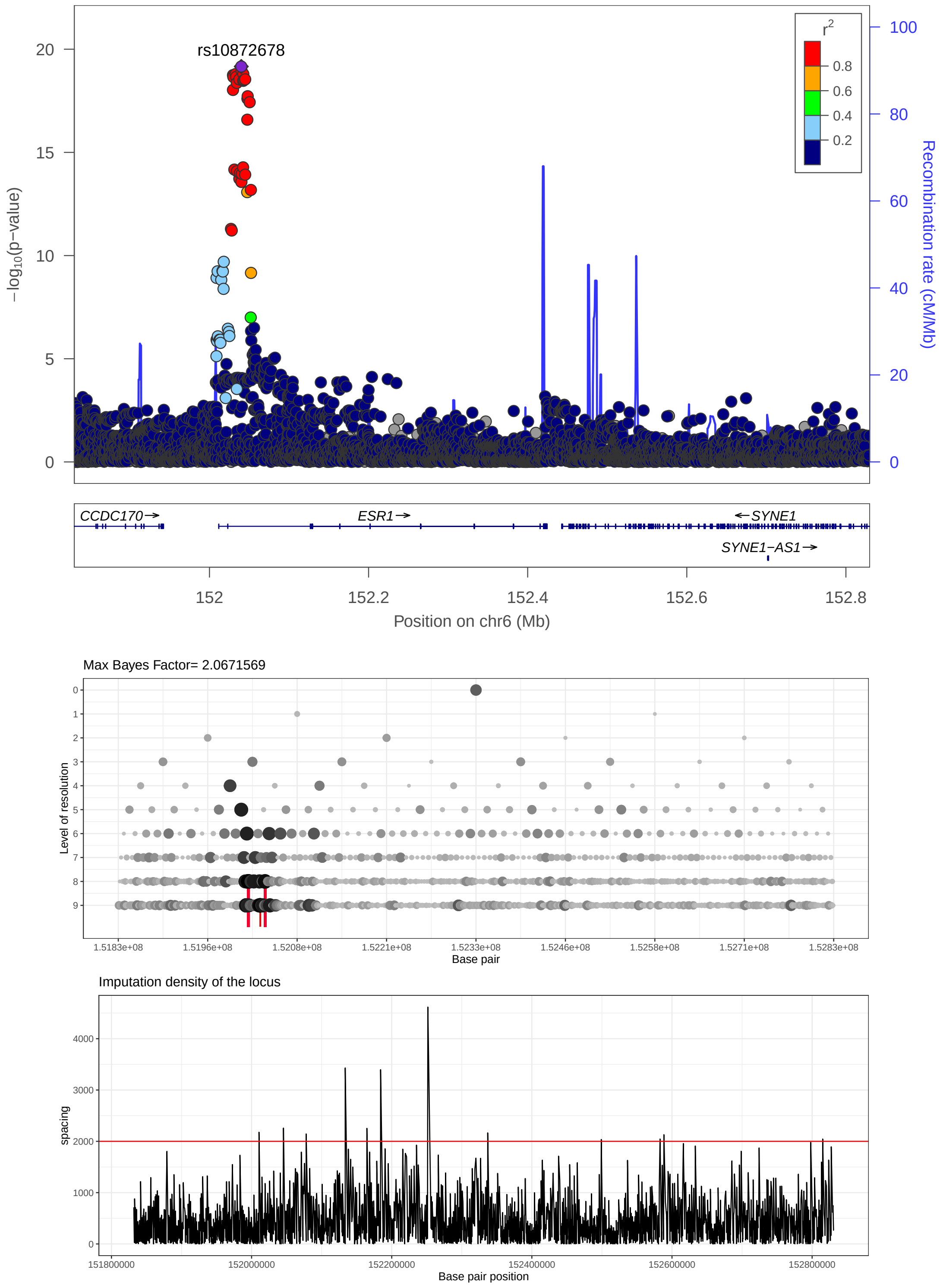


Figure 10: The *ESR1* locus not detected on chromosome 6. The upper panel is a LocusZoom plot of the locus from the summary data from the Horikoshi *et al.* paper [HBD+16].

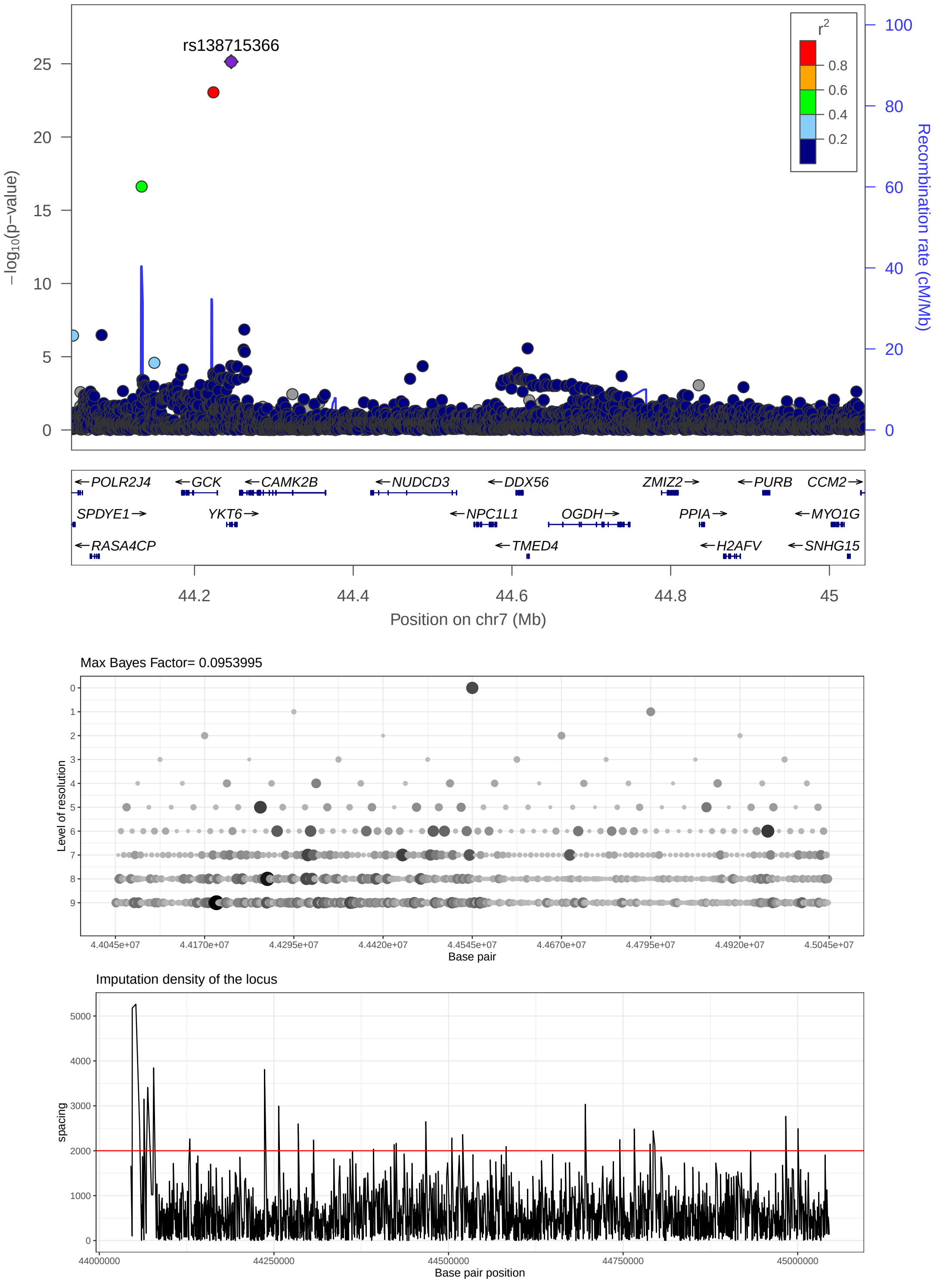


Figure 11: The *YTK6*-*GCK* locus not detected on Chromosome 7. The upper panel is a LocusZoom plot of the locus from the summary data from the Horikoshi *et al.* paper [HBD+16]t.

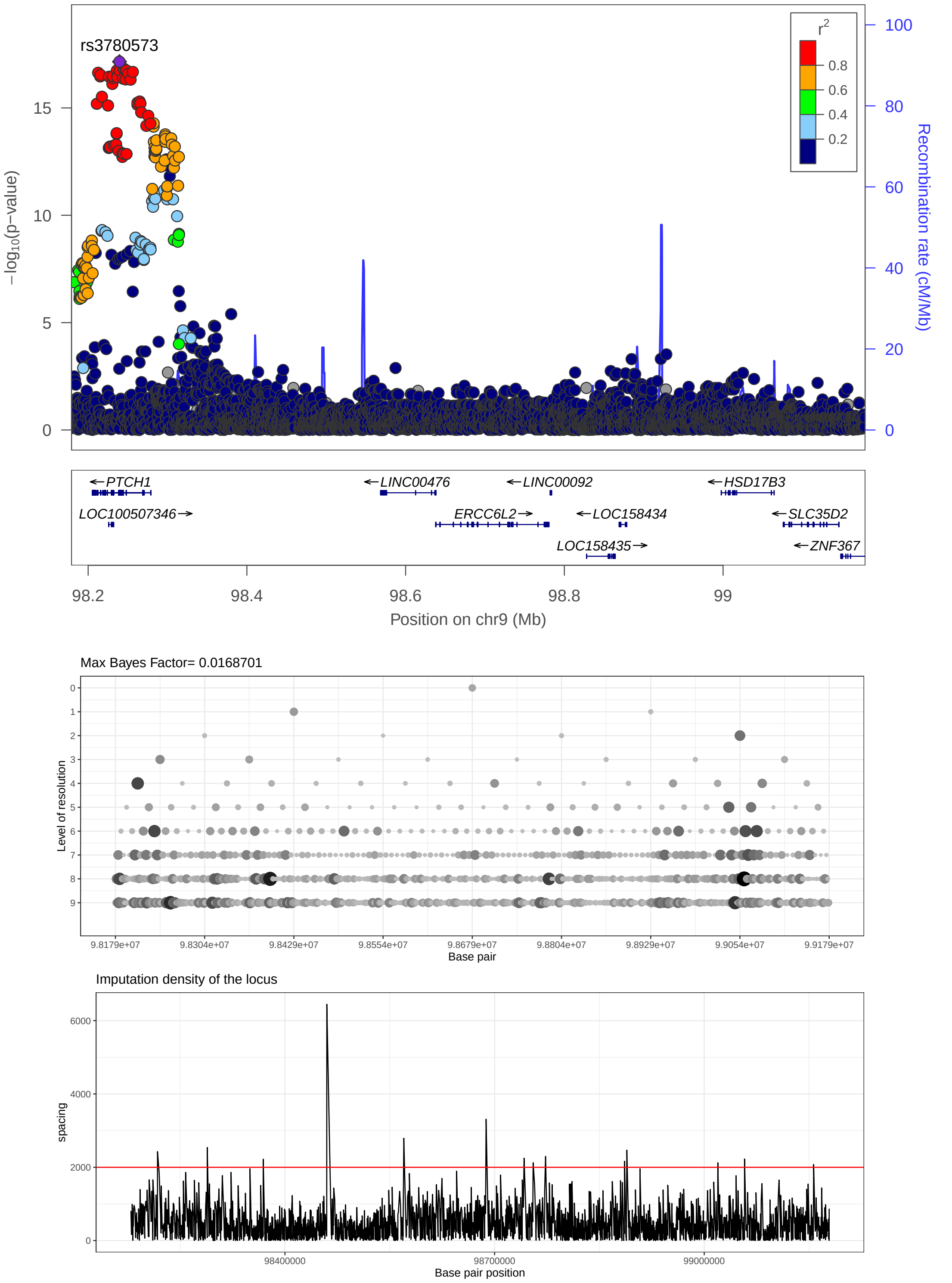


Figure 12: The *PTCH1* locus not detected on chromosome 9. The upper panel is a LocusZoom plot of the locus from the summary data from the Horikoshi *et al.* paper [HBD+16].

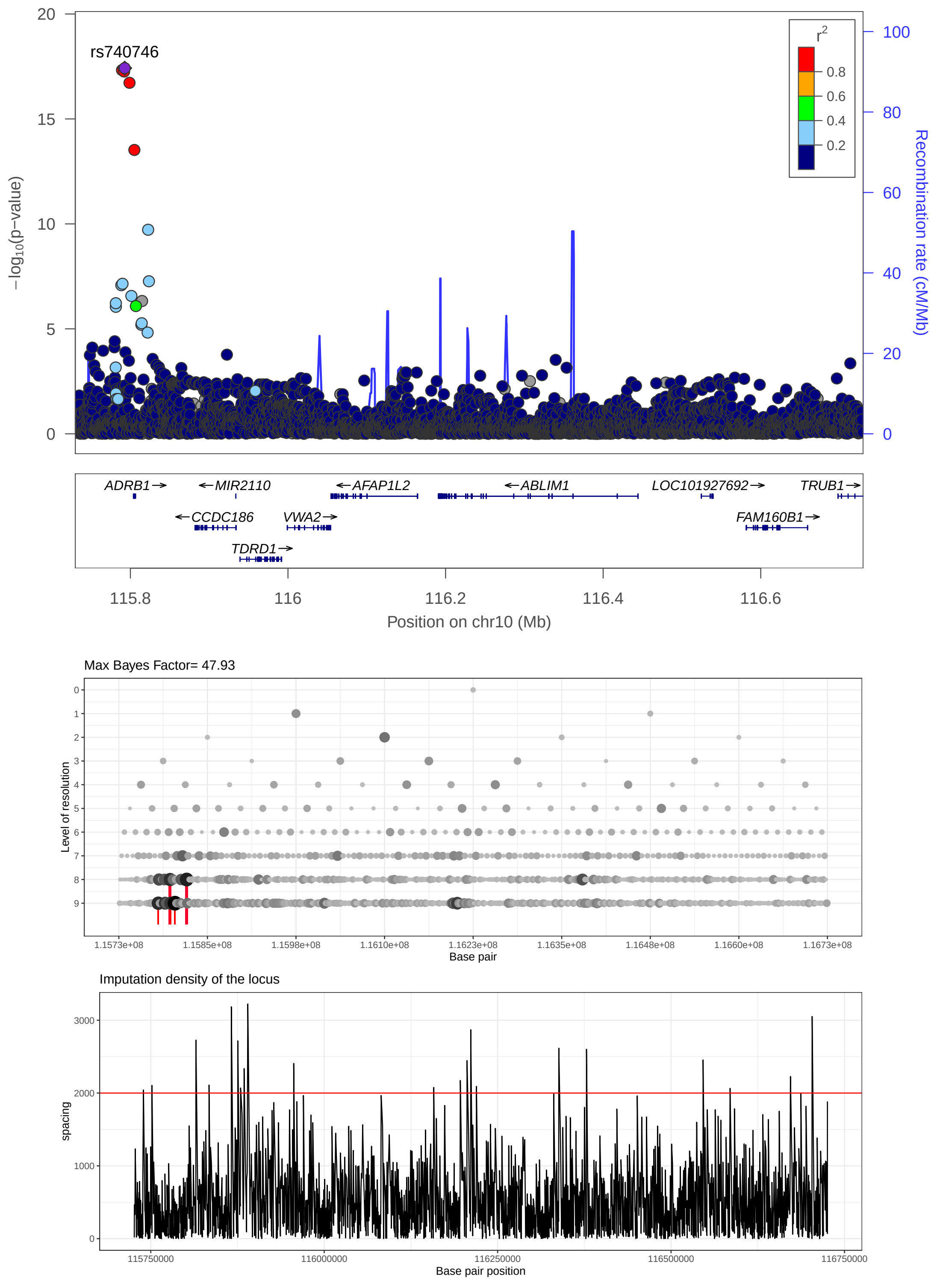


Figure 13: The *ADBR1* locus not detected on chromosome 10. The upper panel is a LocusZoom plot of the locus from the summary data from the Horikoshi *et al.* paper [HBD+16].

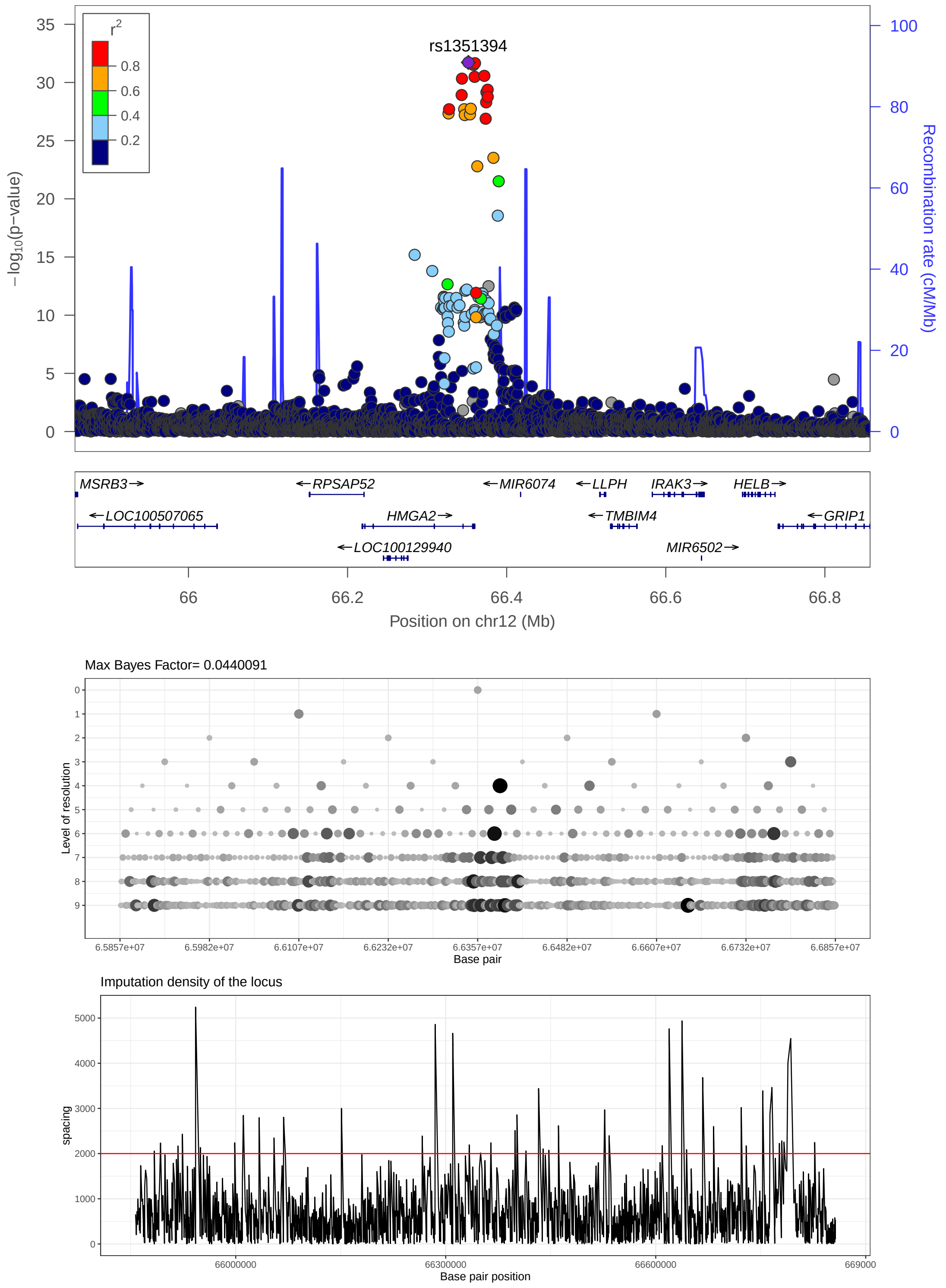


Figure 14: The *HMGA2* locus not detected on chromosome 12. The upper panel is a LocusZoom plot of the locus from the summary data from the Horikoshi *et al.* paper [HBD+16].

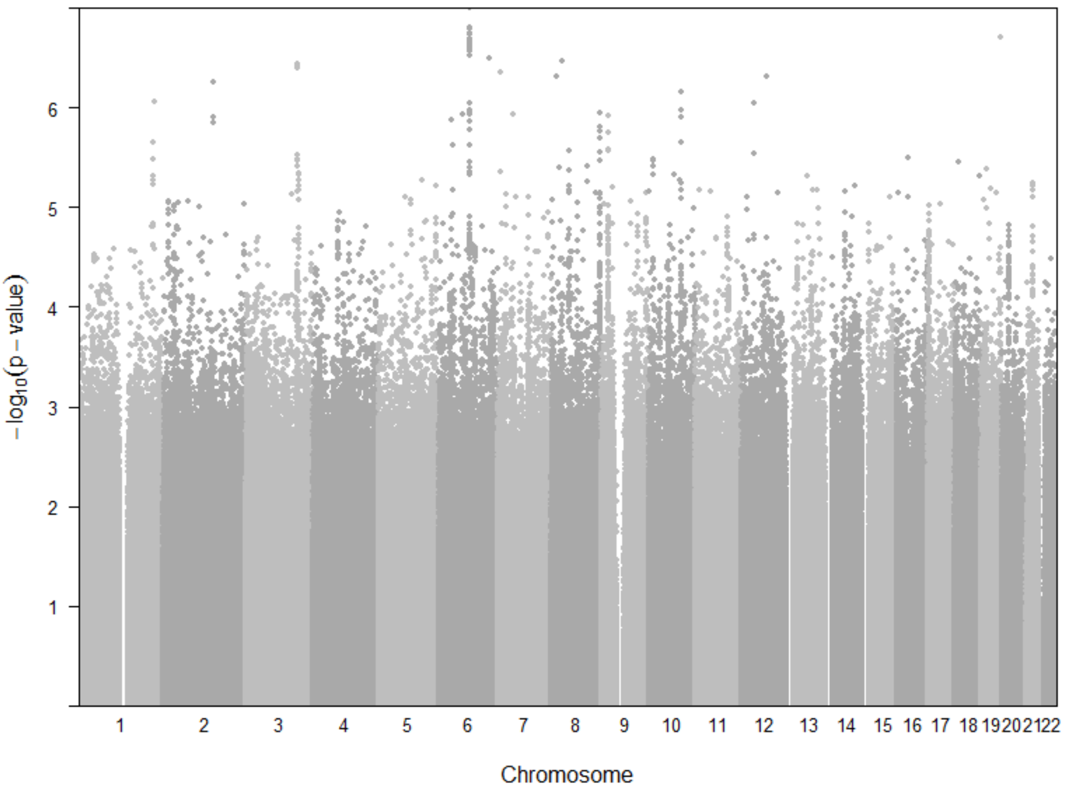


Figure 15: Manhattan plot for the GWAS based on the single-SNP modeling applied to the MoBa dataset.
